# Reticuloendothelial System Function Following Acute Liver Failure Induced by 90% Hepatectomy in the Rat

**DOI:** 10.1155/1993/27630

**Published:** 1993

**Authors:** Xiangdong Wang, Roland Andersson, Jinwen Ding, Lars Norgren, Stig Bengmark

**Affiliations:** Department of Surgery Lund University Lund S-221 85 Sweden

## Abstract

Sepsis and bacterial infections are frequent complications of acute liver failure and following major liver resection. The mechanisms underlying this phenomenon are unclear. In this study, RES function and blood clearance of radiolabelled E. coli was immediately impaired following 90% hepatectomy, although the reduction in liver volume resulted in an increase in splenic (temporary) and pulmonary (persisting) uptake. A significant correlation between liver function and host RES function was observed. The uptake capacity of the RES in the liver remnant and spleen did not correlate with subserosal blood flow. The uptake in the brain gradually increased with time, paralleling an increased leakage across the blood-brain barrier. Thus, a significantly impaired RES function resulted from experimental 90% hepatectomy-induced acute liver failure, which might explain the high incidence of septic events in the clinical situation.


*HPB Surgery*, 1993, Vol. 6, pp. 151-162
Reprints available directly from the publisher
Photocopying permitted by license only

© 1993 Harwood Academic Publishers GmbH
Printed in the United States of America

### RETICULOENDOTHELIAL SYSTEM FUNCTION FOLLOWING ACUTE LIVER FAILURE INDUCED BY 90% HEPATECTOMY IN THE RAT

XIANGDONG WANG, ROLAND ANDERSSON, JINWEN DING, LARS NORGREN and STIG BENGMARK

*Department of Surgery, Lund University, Lund, Sweden*

*(Received 12 May 1992)*

Sepsis and bacterial infections are frequent complications of acute liver failure and following major liver resection. The mechanisms underlying this phenomenon are unclear. In this study, RES function and blood clearance of radiolabelled E. coli was immediately impaired following 90% hepatectomy, although the reduction in liver volume resulted in an increase in splenic (temporary) and pulmonary (persisting) uptake. A significant correlation between liver function and host RES function was observed. The uptake capacity of the RES in the liver remnant and spleen did not correlate with subserosal blood flow. The uptake in the brain gradually increased with time, paralleling an increased leakage across the blood-brain barrier. Thus, a significantly impaired RES function resulted from experimental 90% hepatectomy-induced acute liver failure, which might explain the high incidence of septic events in the clinical situation.

KEY WORDS: Acute liver failure, hepatectomy, reticuloendothelial system, E. coli, liver function, blood flow

### INTRODUCTION

Acute liver failure is a serious complication of, for example, major liver resection[1,2] and is a frequent constituent of multiple organ failure[3-5], which is still associated with a high mortality. The liver represents the major part of the reticuloendothelial system (RES) and Kupffer cells have been reported to account for at least 80% of RES function[6,7]. The importance of the RES is clear – 90% of patients with acute liver failure have bacteriological or clinical evidence of infection[8]. The changes in RES function in the early period of acute liver failure have, however, scarcely been investigated and thus little is known about the pathophysiology that might preceed the occurrence of septic complications.

In the present study we aimed at describing the changes in RES function, including organ distribution and clearance of bacteria, occurring in a model of acute liver failure in the rat, induced by 90% hepatectomy, which untreated results in a 100% mortality within 24-48 hours[9]. We also wanted to investigate the possible correlation between RES function and hepatic function, and between the capacity of bacterial uptake and blood flow in the liver remnant and the spleen.

Address correspondence to: Roland Andersson, M.D, Ph.D, Department of Surgery, Lund University, S-221 85 Lund, Sweden

### MATERIALS AND METHODS

### *Animals*

Eighty-two adult male Sprague-Dawley rats (Anticimex, Stockholm, Sweden), weighing 250-300 gram, were used for the experiments. The animals had standard rat chow ($R_3$, Astra Ewos, Södertälje, Sweden) and tap water ad libitum. The rats were allowed to acclimatize to our laboratory conditions for 4-6 days and were subjected to a regime of 12 hours day/night cycle in mesh stainless-steel cages (3 rats/cage) at a constant room temperature of 22 °C.

### *Surgical Procedures*

The 90% subtotal hepatectomy procedure was a slight modification of what has previously been described in the literature[9,10]. The total rat liver weight was estimated before the operation as 4.5% of the body weight[11]. Under light ether anesthesia, a midline celiotomy with removal of the xiphoid was performed. The left, median and right upper lobes and the right lower lobe or most of it were in general included in the 90% hepatectomy, thus leaving only the caudate lobe and in a few instances also parts of the right lower lobe. After the hepatectomy, a coagulant (Spongostan, Ferrosan, Denmark) was applied on the cut surface.

The control group consisted of sham operation using a similar midline celiotomy with separation of the liver from the surrounding structures without hepatectomy.

### *Radiolabelling of E. Coli*

E. coli (serotype 046:K1:H31) was cultured overnight in Todd-Hewitt broth, heat-killed at 88 °C for 15 min and washed 3 times in phosphate-buffered saline (PBS, Sigma Chemical Co., St. Louis, USA, including 0.03 M phosphate, 0.12 M saline, pH 7.2) and thereafter suspended in PBS. The bacterial labelling with $I^{125}$ was performed according to the protein-labelling method of McConahey and Dixon,[12] using choloramine-T as the oxidizing agent. This labelling was followed by three additional washings in PBS and centrifugations (1800 rpm for 10 min), after which the suspension was frozen at -20 °C, and thawed immediately before use. The radioactivity was approximately $1.5 \times 10^7$ counts/ml bacterial suspension.

### *Blood Clearance and Organ Distribution of Radiolabelled E. Coli*

Blood clearance and organ distribution were studied in 24 rats allocated into four groups of 6 animals each: controls subjected to sham celiotomy, and studies performed immediately (0), 6 and 12 hours after 90% hepatectomy. The femoral artery (OD 0.75 mm, Portex, Kent, England) and vein (OD 0.94 mm, Dow Corning, Michigan, USA) were cannulated under ether anesthesia immediately, 6 or 12 hours after hepatectomy. 200 $\mu$l of the labelled E. coli suspension ($3.0 \times 10^6$ cpm) was injected into the femoral vein. Arterial blood samples (150 $\mu$l) were taken immediately before and 2, 5, 8, 10 and 15 min after the intravenous injection of radiolabelled E. coli. The animals were sacrificed directly after the 15 min sample. Standardized parts of the various organs (the caudate liver lobe, the upper part of the spleen, the right lower lobe of the lungs, the middle part of the left kidney, the thymus, the left ventricle wall of the heart, the pancreatic tail, the femoral muscle, the epiphysis of the femoral bone and the parietosphenoid lobe of the brain, respectively) were removed and the weight of the individual organs were determined. The radioactivity of all specimens (blood and organ specimens) was measured using a 1260 Multigamma counter (LKB, Stockholm, Sweden) and expressed as $I^{125}$ cpm per gram tissue or ml blood. The radioactivity was calculated as cpm/gram tissue, and also presented in percentage comparing the ten different organs studied at the different time points following hepatectomy.

Blood clearance of $I^{125}$ E. coli was described by calculating the elimination rate constant (k-value) using the method of least square for each animal and the k (mean) $\pm$ SEM for each group[13].

The effectiveness of the blood-brain barrier to exclude the radiolabelled E. coli from passing was estimated according to what has been described by Daniel *et al.*[14,15], where the constant of transfer (Kd) is a measure of the permeability from blood to the central nervous system.

*Mortality and Liver Function Tests*

Death occurred in 10 rats subjected to 90% hepatectomy prior to the 6 hour intervals without challenge with bacteria or blood sampling. In the group of 24 animals, 500 $\mu$l of blood was taken from the tail vein one day prior to hepatectomy, immediately after and at 6 and 12 hours following 90% hepatectomy ($n = 6$ at each time point) for the analysis of plasma levels of bilirubin, alkaline phosphatase (ALP), aspartate aminotransferase (ASAT) and alanine aminotransferase (ALAT). Following each blood sampling, 500 $\mu$l of sterile saline was injected into the tail vein.

*Evaluation of Host RES Function*

Body uptake rate was used to evaluate total RES function. A theoretical standard uptake value for the rat was defined by the mean values of the total uptake of four main RES organs (liver, spleen, lungs and kidneys) in each rat subjected to sham operation. The body uptake rate was defined as the total uptake in the four organs in the various experimental groups compared with the theoretical standard uptake value.

*Laser Doppler Flowmetry*

Subserosal blood flow in the liver remnant and the spleen was measured in 24 animals using a Laser Doppler flowmeter (Periflux, Pf-2.; Perimed, Sweden). This instrument contains a laser that generates monochromatic light of 632.8 nm wavelength with a penetration depth of 1.0 mm. The fiberoptic guide system is 13.5 cm in length and has a flow probe 0.5 mm in diameter with a holder 1.8 cm in diameter. The end of the flow probe was gently placed on the surface of the organ to be measured and held there to obtain flux value (% of initial value), which was used to express the degree of blood flow in the tissue under the serosal surface. Visible vessels were avoided to obtain more accurate tissue flow values[16]. Six points were chosen in each organ and each measured with 100 X 4 KHZ for 3 seconds. The mean $\pm$ SEM was used to express the subserosal blood flow in the liver and the spleen at the various time points.

*Statistical Methods*

Student's *t*-test was used for statistical analysis of liver function tests, blood clearance of radiolabelled E. coli, uptake of radiolabelled E. coli per gram tissue, constant of transfer of the blood-brain barrier and phagocytic index. Chi-square was used for the analysis of the percentage distribution of the uptake of labelled E. coli between the various organs. Kendall correlation was used to evaluate the correlation between liver function tests and RES function, and between blood flow and uptake capacity of the liver remnant and the spleen. Statistical analysis of all blood flow values was performed by using Student's *t*-test for unpaired data. Values are expressed as mean ± SEM.

### RESULTS

Clinical signs of acute liver failure started to develop within 6 hours following the 90% hepatectomy, including diminished motor activity, lethargy, piloerection, trembling, somnolence or even coma. Mortality was 30% six hours following hepatectomy and 50% after 12 hours. Plasma levels of standard liver function tests significantly increased 6 and 12 hours after 90% hepatectomy as compared to preoperative levels ($p<0.05$ and $p<0.01$; Table 1).

The significantly impaired blood clearance of radiolabelled E. coli seen immediately after 90% hepatectomy, persisted 6 and 12 hours after operation ($p<0.01$; Table 2), without any difference between the different time points.

**Table 1** Liver function tests at different time points following 90% hepatectomy in the rat

| | *Bilirubin (mg/l)* | *ALAT μKat/l* | *ASAT μKat/l* | *ALP μKat/l* |
|---|---|---|---|---|
| Sham-operation | 8.8±2.4 | 3.6±0.9 | 7.4±2.5 | 10.2±3.1 |
| 90%H-0 hour | 11.3±4.7 | 4.5±2.3 | 8.7±3.4 | 12.5±2.7 |
| 90%H-6 hours | 20.3±4.5* | 10.6±4.4* | 18.5±5.1* | 21.4±4.4* |
| 90%H-12 hours | 40.8±6.3** | 20.3±6.8** | 34.7±7.6** | 41.2±5.8** |

The plasma levels of liver enzymes and bilirubin in rats were analyzed immediately (0 hour), 6 and 12 hours after 90% hepatectomy (90% H). The values are mean±SEM. Each group included six animals. * stands for probability level $p<0.05$ as compared to the group with sham-operation.

**Table 2** Blood clearance of radiolabelled E. coli at different times following 90% hepatectomy in the rat, expressed as the elimination rate constant

| *Group* | *K-value* | *P** |
|---|---|---|
| Sham-operation | −0.100088±0.003943 | |
| 90%H-0 hour | −0.041909±0.005269 | <0.01 |
| 90%H-6 hours | −0.042797±0.03612 | <0.01 |
| 90%H-12 hours | −0.050855±0.008539 | <0.01 |

K-value expresses the elimination rate constant immediately (0 hour), 6 and 12 hours after 90% hepatectomy (90% H). The values are mean±SEM. Each group included six animals. * stands for probability level as compared to sham operation.

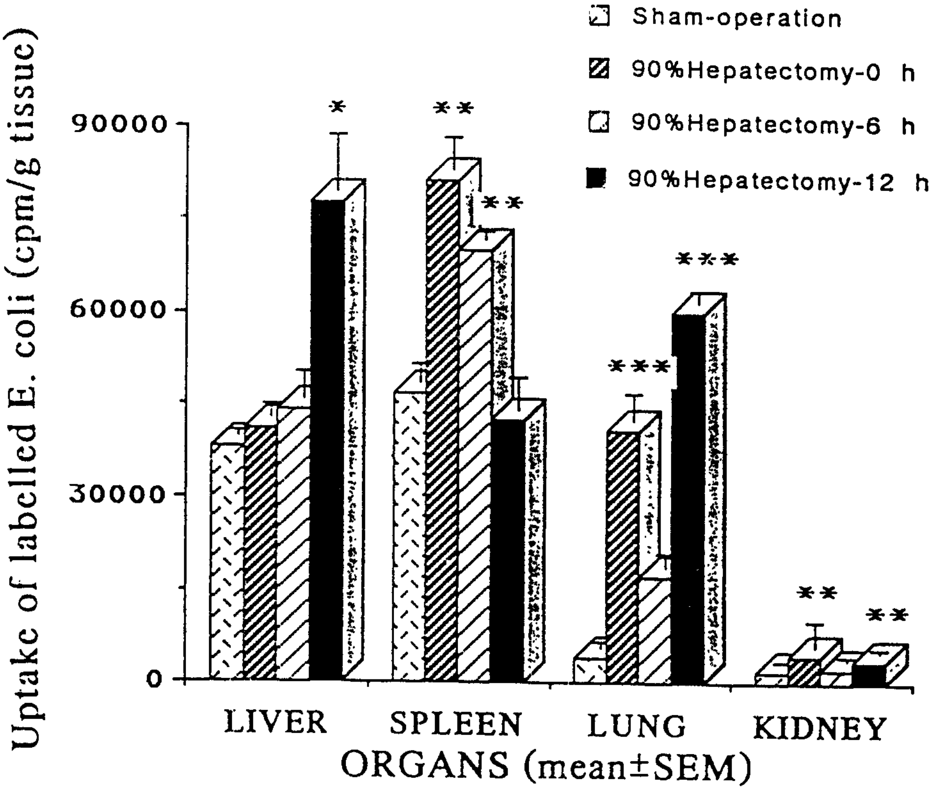

**Figure 1** Organ distribution of E. coli immediately (0 h), 6 hours (6h) and 12 hours (12 h) following 90% hepatectomy in the rat. Values are mean ± SEM. Each group included six animals. * indicates probability level $p<0.05$ as compared to sham operation; **indicates probability level $p<0.01$ as compared to sham operation.

Figure 1 shows the uptake of labelled bacteria in the liver, spleen, lungs and kidneys in controls and in animals subjected to 90% hepatectomy immediately (0), 6 and 12 hours after hepatectomy, expressed as cpm/gram tissue. As can be seen, liver uptake was augmented, 12 hours after the operation it was significantly higher than in controls ($p<0.05$). The initial increase in splenic uptake ($p<0.01$) gradually decreased to similar levels as in the control group. The increase in pulmonary uptake ($p<0.001$) after the hepatectomy, however, tended to persist, as did the increase in renal uptake ($p<0.01$), though radioactive levels in this particular organ were low. The uptake of radiolabelled bacteria in the brain gradually increased with time after subtotal hepatectomy ($p<0.01$), paralleling the significantly higher constant of transfer (Kd) at 6 and 12 hours after hepatectomy ($p<0.01$; Figure 2).

The percentage distribution of the total uptake of radiolabelled bacteria in the liver, spleen, lungs and kidneys is shown in Table 3. The liver dominates the uptake prior to hepatectomy, but is then reduced to levels similar to that found in the

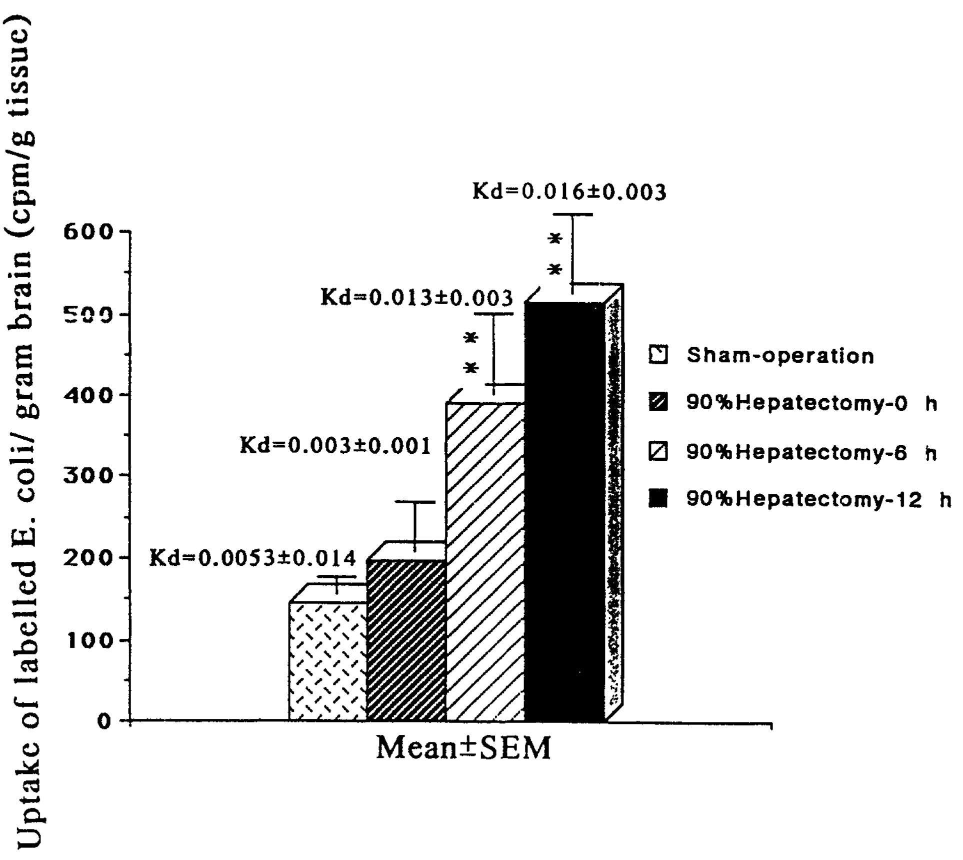

**Figure 2** Uptake of radiolabelled E. coli in the brain and the constant of transfer (Kd) immediately (0 h), 6 hours (6 h) and 12 hours (12 h) following 90% hepatectomy in the rat. Values are mean ± SEM. Each group included six animals. **indicates probability level $p < 0.01$ as compared to sham operation.

**Table 3** The percentage distribution of the total uptake of radiolabelled E. coli in the liver, spleen, lungs and kidneys at different time points following 90% hepatectomy in the rat

| | *Liver* | *Spleen* | *Lungs* | *Kidneys* |
|---|---|---|---|---|
| Sham-operation | 87.7±0.4 | 9.9±0.4 | 1.5±0.2 | 0.9±0.1 |
| 90%H-0 hour | 25.4±1.4** | 31.1±2.2** | 34.5±2.2** | 7.8±0.6* |
| 90%H-6 hours | 37.4±4.8** | 41.5±4.7** | 16.5±2.6* | 4.3±0.6 |
| 90%H-12 hours | 44.2±4.2** | 14.1±2.4 | 36.9±3.6** | 4.9±0.3 |

The percentage distribution means uptake in each organ in proportion to the total uptake in all four organs immediately (0 hour), 6 and 12 hours after 90% hepatectomy (90% H). The values are mean±SEM. Each group included six animals. * and ** stand for probability level $p<0.05$ and $p<0.01$, respectively, as compared to the group with sham-operation.

**Table 4** Percentage distribution of the uptake of radiolabelled E. coli per gram tissue in ten various organs/tissues following 90% hepatectomy in the rat

| | *Sham-operation* | *90%H-0 h* | *90%H-6 h* | *90%H-12 h* |
|---|---|---|---|---|
| Liver | 39.3±4.3 | 24.0±1.7* | 33.2±4.2 | 39.9±3.7 |
| Spleen | 51.5±4.6 | 46.6±1.7 | 49.2±6.8 | 28.6±3.2* |
| Lung | 4.2±0.5 | 23.4±2.0** | 12.8±3.8* | 29.9±3.4** |
| Kidney | 1.7±0.1 | 2.4±0.1* | 0.8±0.1* | 1.8±0.1 |
| Bone | 0.6±0.1 | 0.9±0.1 | 1.0±0.1 | 1.5±0.04* |
| Pancreas | 0.8±0.1 | 1.0±0.1 | 1.7±0.2 | 0.8±0.1 |
| Heart | 0.5±0.04 | 0.7±0.1 | 0.5±0.04 | 1.5±0.3* |
| Thymus | 0.7±0.1 | 0.5±0.03 | 0.3±0.03 | 0.5±0.1 |
| Muscle | 0.4±0.1 | 0.3±0.03 | 0.3±0.1 | 0.3±0.04 |
| Brain | 0.2±0.03 | 0.1±0.02 | 0.3±0.03 | 0.3±0.04 |

The percentage distribution of the uptake per gram tissue in each organ/tissue in proportion to the total uptake in ten various organs/tissues measured immediately (0 h), 6 (6 h) and 12 hours (12 h) after 90% hepatectomy (90% H). The values are mean±SEM. Each group included six animals. * and ** stand for probability levels $p<0.05$ and $p<0.01$, respectively, as compared to the group with sham-operation.

spleen and lungs. In Table 4, it can be seen that among the various organs, the highest uptake is within the liver, spleen and lungs, i.e. the major organs of the RES, with a gradual increase in the uptake/g liver tissue to sham levels in time after 90% hepatectomy, this follows an initial decrease.

RES function significantly decreased following hepatectomy as compared to sham operation ($p<0.01$, Figure 3). The body uptake rate of radiolabelled E. coli decreased immediately after 90% hepatectomy, only being 30 ± 2% of the theoretical value (range between 26 and 35%). A minor, though not significant, increase in the body uptake rate occurred at 6 and 12 hours as compared to immediately after hepatectomy. Plasma bilirubin and liver enzyme levels showed an inverse correlation with the body uptake rate ($r = -0.336$ and $r = -0.280$, respectively, $p<0.05$).

The subserosal blood flow values in the caudate liver lobe and the spleen in rats with or without 90% hepatectomy-induced ALF are shown in Figure 4. Blood flow values in the liver remnant significantly decreased immediately after the 90% hepatectomy (33.0 ± 3.3, $p<0.001$), as well as 6 hours after hepatectomy (57.3 ± 2.8, $p = 0.002$), but were not different 12 hours postoperatively (67.8 ± 5.3, $p = 0.466$), as compared with controls (71.7 ± 2.4). A significant recovery of blood flow values was gradually observed at 6 and 12 hours as compared to immediately after 90% hepatectomy ($p<0.001$). Blood flow values in the spleen significantly decreased immediately after 90% hepatectomy (24.1 ± 2.0, $p<0.001$), and returned to control levels 6 hours postoperatively (37.4 ± 1.4, $p = 0.553$) and then diminished again 12 hours after hepatectomy (12.7 ± 1.6, $p<0.001$), as compared with controls (40.5 ± 2.0). Splenic blood flow values 12 hours post-hepatectomy were also singificantly lower than immediately after 90% hepatectomy ($p = 0.003$). No significant correlation between the uptake of labelled bacteria and blood flow in the liver remnant and spleen was found ($r = 0.4$ and $r = 0.2$, $p>0.05$, respectively).

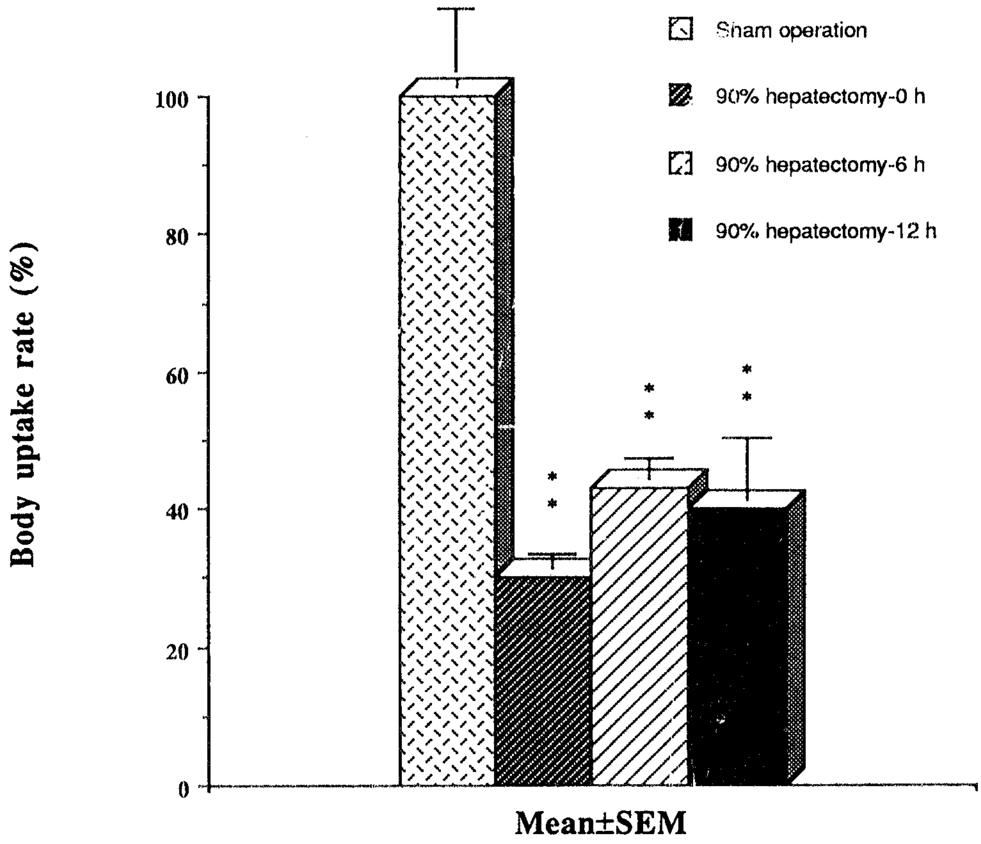

**Figure 3** Body uptake rate of radiolabelled E. coli immediately (0 h), 6 hours (6 h) and 12 hours (12 h) following 90% hepatectomy in the rat. Values are mean ± SEM. Each group included six animals. **indicates probability level $p < 0.01$ as compared to sham operation.

### COMMENT

Bacterial infection and sepsis are frequent findings accompanying the early course of acute liver failure[8]. The pathophysiological mechanism(s) explaining the bacterial complications in acute liver failure are not clear, but dysfunction in host defense mechanisms have been suggested, such as impaired function of Kupffer cells and polymorphonuclear leukocytes[17,18] and reduced blood levels of fibronectin, opsonins and chemoattractants, including components of the complement cascade system[19]. The present study supports previous reports, showing that the liver vastly dominates the RES total capacity, being responsible for 80-90% of RES function[6,7,13].

RES function has been well recognized as one of the main constituents of host immune function. Traditionally, it was thought that RES function in the individual organs parallels the host's general immune responses[20]. In 90% hepatectomy-

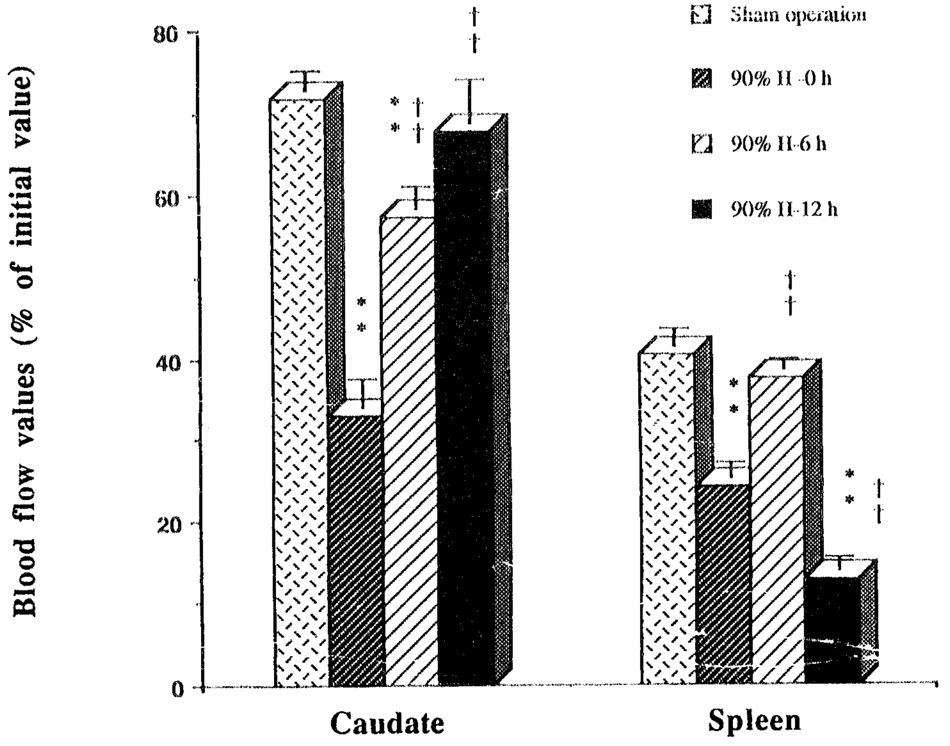

**Figure 4** The subserosal blood flow values in the caudate liver lobe and spleen immediately (0 h), 6 hours (6 h) and 12 hours (12 h) following 90% hepatectomy in the rat. Values are mean ± SEM. Each group included six animals. **indicates probability level $p<0.01$ as compared to sham operation; induced acute liver failure, however, changes of host RES function did not correlate with that in the various organs. The increase of uptake capability of the spleen, lungs and kidneys, and also the liver remnant, appeared to compensate for the generally impaired uptake capacity caused by 90% hepatectomy. The distribution of RES function, seen when comparing whole body and individual organs occurred in the early stage of 90% hepatectomy-induced acute liver failure. This was demonstrated in the present study by the body uptake rate of labelled bacteria representing host RES function and the uptake rate in the individual organs representing organ RES function. These findings show that the changes of RES function in organs or tissues previously reported occurred in order to meet needs with the parallel responses of host immune function[13,21].

Attention has recently been focussed on the association of gut origin bacteremia and hepatic dysfunction. Zarkin *et al.*[22] reported that 52% of patients with Streptococcus bovis endocarditis and 57% of those with S. bovic bacteremia had liver disease identified by plasma bilirubin and serum transaminase levels or by histologic findings on liver biopsies. They hypothesized that liver diseases might lead to an impairment of hepatic RES function, which might play a major role in the development of S. bovis bacteremia or endocarditis. We have previously demonstrated the gradual development of translocation of enteric bacteria from the gut to the mesenteric lymph nodes or systemic circulation following various degrees of liver resection[23]. In the present study, the increase in plasma bilirubin and liver enzyme levels followed the reduced host RES function, indicating that hepatic dysfunction of various causes in patients could be suspected to be followed by a decrease in host RES function. Deitch *et al.*[24] recently demonstrated that translocating enteric bacteria impaired systemic immunity, using the E. coli C25 monoassociation model. We have also proved that bacterial translocation of gut origin in rats with 90% hepatectomy-induced acute liver failure occurred 2 hours following 90% hepatectomy (unpublished data). Our present results show that host RES function was impaired immediately following 90% hepatectomy, prior to bacterial translocation from the gut to the mesenteric lymph nodes or bloodflow changes could be demonstrated. Thus, impaired systemic immunity might preceed bacterial translocation of gut origin. Hemodynamic changes caused by subtotal liver resection could be considered as a possible factor influencing organ RES function. Portal hypertension has been shown to be one major complication of acute or chronic liver failure[25]. In the present study, subserosal blood flow in the liver remnant and the spleen decreased immediately following 90% liver resection, maybe due to blood retention induced by temporary portal hypertension. However, RES function in the liver remnant and the spleen did not prove to depend on blood flow changes.

A direct influence on RES capacity in the early period of acute liver failure induced by 90% hepatectomy could be demonstrated, as blood clearance of radiolabelled E. coli was significantly impaired. It is evident that this decreased clearance mainly seems to be the result of a marked decrease in the early, otherwise highly potent, clearance phase. Such a decrease in host defense causing a diminished capacity to clear bacteria from the systemic circulation and different organs might contribute to the explanation of the high incidence of sepsis and infectious complications associated with major liver resection and acute liver failure in the clinical situation. The primary source of the pathogenetic bacteria and the way they reach the infectious/abscess site intraabdominally, e.g. following major liver resection, is not fully understood, but it is known that the majority of cultured bacteria in patients with abdominal sepsis/abscess following major liver resection seem to be of gut origin[23] and the occurrence of an impairment in host defense including an increase in gut translocation of bacteria induced by major hepatectomy is possible. Even though RES function in general decreased following 90% hepatectomy, the uptake of bacteria per gram tissue in the liver remnant significantly increased 12 hours after resection. This augmentation of Kupffer cell function support previous findings that activation of Kupffer cell function occurs in the regenerating liver following liver resection[21,26], this maybe a result of the regenerative process, regulated by hepatotrophic factors or induced by massive hepativ necrosis with release of several cytokines, which might play a major role in the development of endothelial cell destruction and fibrin deposition in the hepatic sinusoids[26]. On the other hand, cachectin/tumor necrosis factor, released from the activated Kupffer cells or macrophages, can mediate and participate in the development of multiple organ failure[27].

A general redistribution of the uptake of radiolabelled bacteria in the various organs responsible for the major capacity of RES function could be noted following

90% hepatectomy. A temporary increase in splenic uptake was noted, while the increase in pulmonary uptake persisted during the study period, a finding that is similar to other conditions where RES function is impaired, e.g. experimental peritonitis[28-30] or blocking of the RES with macromolecules[13]. This might lead to pulmonary trapping of macromolecules, bacteria and particulate matter resulting in adult respiratory distress syndrome[31,32]. The renal increase in bacterial uptake seen following hepatectomy might be one of the reasons for the hepatorenal failure which can be seen in acute liver failure with a coexisting systemic bacteremia or endotoxemia[8]. The gradual increase in uptake of radiolabelled bacteria in the brain and the increased leakage over the blood-brain barrier probably goes together with a general increased leakage to the brain of various toxic substances like ammonia, mercaptans and others.

In conclusion, RES function was impaired following the induction of acute liver failure by 90% hepatectomy, with a concomitant redistribution of organ uptake.

### *References*

1. Iwatsuki, S. & Starzi, T. (1988) Personal experience with 411 hepatic resection. *Ann.Surg.*, **208**, 421–434
2. Nagao, T., Inoue, S., Goto, S., *et al.* (1987) Hepatic resection for hepatocellular carcinoma. *Ann.Surg.*, **205**, 33--40
3. Polk, H.C. & Shields, C.L. (1977) Remote organ failure: a valid sign of occult intra-abdominal infection. *Surgery*, **81**, 310–313
4. Eiseman, B., Beart, R. & Norton, L., (1977) Multiple organ failure. *Surg.Gynecol.Obstet.*, **144**, 323–326
5. Cerra, F.B., Siegel, J.H. & Border, J.R. (1979) The hepatic failure of sepsis: cellular vs substracte. *Surgery*, **86**, 409–421
6. Biozzi, G. & Stiffel, C. (1965) The physiopathology of the reticuloendothelial cells of the liver and the spleen. In: Popper, H. and Shaffner, F. (Eds): *Progress in liver diseases* pp. 166–169. New York: Grune & Stratton
7. Biozzi, G., Benacerraf, B. & Halpern, B.N. (1953) Quantitative study of the granulopoetic activity of the reticuloendothelial system II. A study of the kinetics of the granulopoetic activity of the RES in relation to the dose of carbon injected. Relationship between the weight of the organs and their activity. *Br.J.Exp.Pathol.*, **34**, 441–448
8. Rolando, N., Harvey, F. & Brahm, J. (1990) Prospective study of bacterial infection in acute liver failure: An analysis of fifty patients. *Hepatology*, **11**, 49–53
9. Wang, X.D., Ar'Rajab, A., Ahrén, B., Andersson, R. & Bengmark, S. (1991) The effect of pancreatic islets on transplanted hepatocytes in the treatment of acute liver failure in rats. *Res.Exp.Med.*, **191**, 429–435
10. Demetriou, A.A., Reisner, A., Sanchez, J., Levenson, S.M., Moscioni, A.D. & Chowdhury, J.R. (1988) Transplantation of microcarrier-attached hepatocytes into 90% partially hepatectomized rats. *Hepatology*, **8**, 1006–1009
11. Wang, S.R., Renand, G., Linfante, J., Catala, D. & Infante, R. (1985) Isolation of rat hepatocytes with EDTA and their metabolic function in primary culture. *In Vitro*, **21**, 526–532
12. McConahey, P.J. & Dixon, F.J. (1966) A method of trace iodination of proteins for immunological studies. *Arch.Allergy*, **29**, 185–189
13. Andersson, R. & Bengmark, S. (1989) Influence of dextran on blood clearance and organ distribution of radiolabelled E. coli and on survival in experimental E. coli septicemia. *Acta Chir.Scand.*, **155**, 151–154
14. Daniel, P.M., Lam, D.K.C. & Pratt, E. (1981) Changes in the effectiveness of the blood-brain and blood spinal cord barriers in experimental allergic encephalomyelitis. *J.Neurol.Sci.*, **52**, 211–219
15. Daniel, P.M., Lam, D.K.C. & Pratt, E. (1983) Relation between the increase in the diffusional permeability of the blood-central nervous system barrier and other changes during the development of experimental allergic encephalomyelitis in the Lewis rat. *J.Neurol.Sci.*, **60**, 367–376
16. Arvidsson, D., SVensson, H. & Haglund, U. (1988) Laser-Doppler Flowmetry for estimating liver blood flow. *Am.J.Physiol.*, **254**, G471–478

17. Canalese, J., Gove, C.D., Gimson, A.E.S., Wilkinson, S.P., Wadle, E.N. & Williams, R. (1982) Reticuloendothelial system and hepatocyte function infulminant hepatic failure. *Gut*, **23**, 265–269
18. Wyke, R.J., Yousif-Kadaru, A.G.M. & Rajkovic, I.A. (1980) Serum stimulatory activity and polymorphonuclear leukocyte movement in patients with fulminant hepatic failure. *Clin.Exp.Immunol.*, **51**, 442–449
19. Wyke, R.J., Raijkovic, I.A., Eddleston, A.L.W.F. & Williams, R. (1980) Defective opsonization and complement deficiency in serum from patients with fulminant hepatic failure. *Gut*, **21**, 643–649
20. Dean, R.T. & Jessup, W. (1985) Mononuclear phagocytes: physiology and pathology. In *Research monographs in cell and tissue physiology*. General editors: Dingle, J.T. and Gordon, J.L. Elsevier Science Publishers B.V.
21. Gross, K., Katz, S., Dunn, S.P., Cikrit, D., Rosenthal, R. & Grosfeld, J.L. (1985) Bacterial clearance in the intact and regenerating liver. *J.Pediatr.Surg.*, **20**, 320–333
22. Zarkin, B.A., Lillemoe, K.D., Cameron, J.L., Effiron, P.N., Magnuson, T.H. & Pitt, H.A. (1990) The triad of Streptococcus bovis bacteremia, colonic pathology, and liver disease. *Ann.Surg.*, **211**, 786–792
23. Wang, X.D., Andersson, R., Soltesz, V. & Bengmark, S. (1992) Bacterial translocation after major hepatectomy in patients and rats. *Arch.Surg.* (in press)
24. Deitch, E.A., Dazhong, X., Qi, L. & Berg, R.D. (1991) Bacterial translocation from the gut impairs systemic immunity. *Surgery*, **109**, 269–276
25. Takenaka, H., Nakao, K., Miyata, M., *et al.* (1990) Hemodynamic study after devasculization procedure in patients with esophageal varices. *Surgery*, **107**, 55–62
26. Fujiwara, K., Ogata, I., Mochida, S., *et al.* (1990) Activated Kupffer cells as a factor of massive hepatic necrosis after liver resection. *Hepato.Gastroenterol.*, **37**, 194–197
27. Tracey, K.J., Fong, Y., Hesse, D.G., *et al.* (1987) Anti-cachecin/TNF monoclonal antibodies present septic shock during lethal bacteraemia. *Nature*, **330**, 662–664
28. Andersson, R., Schalén, C. & Tranberg, K.G. (1991) The influence of bile on growth, clearance and organ uptake of Escherichia in E. coli peritonitis in the rat. *Arch.Surg.*, **126**, 773–777
29. Hansson, L., Jeppson, B., Srinivas, U., Alwmark, A., Christensen, P. & Bengmark, S. (1988) Blood clearance and organ distribution of Escherichia cells during Gram-negative sepsis in the rat. *Surg.Res.Comm.*, **3**, 311–317
30. George, C., Carlet, J., Sobet, A., *et al.* (1980) Circulating immune complexes in patients with gram negative septic shock. *Intensive Care Med.*, **6**, 123–127
31. Horn, J.K., Goldstein, I.M. & Flick, M.R. (1984) Complement and endotoxin-induced lung injury in sheep. *J.Surg.Res.*, **36**, 420–424
32. Rubin, D.B., Wiener-Kronish, J.R., Muray, J.F., *et al.* (1990) Elevated von Willebrand factor antigen is an early plasma predictor of acute lung injury in nonpulmonary sepsis syndrome. *J.Clin.Invest.*, **86**, 474–480

